# Exploration of altered miRNA expression and function in MSC-derived extracellular vesicles in response to hydatid antigen stimulation

**DOI:** 10.3389/fmicb.2024.1381012

**Published:** 2024-03-27

**Authors:** Xin Wang, Wubulikasimu Mijiti, Qiyu Jia, Zhifei Yi, Junchao Ma, Ziyu Zhou, Zengru Xie

**Affiliations:** ^1^Department of Orthopedics and Trauma, The First Affiliated Hospital of Xinjiang Medical University, Ürümqi, Xinjiang, China; ^2^Key Laboratory of High Incidence Disease Research in Xingjiang (Xinjiang Medical University), Ministry of Education, Ürümqi, Xinjiang, China; ^3^Xinjiang Clinical Research Center for Orthopedics, Xinjiang Medical University, Ürümqi, Xinjiang, China

**Keywords:** hydatid disease, extracellular vesicles, antigens, *Echinococcus*, microRNAs

## Abstract

**Background:**

Hydatid disease is caused by *Echinococcus* parasites and can affect various tissues and organs in the body. The disease is characterized by the presence of hydatid cysts, which contain specific antigens that interact with the host’s immune system. Mesenchymal stem cells (MSCs) are pluripotent stem cells that can regulate immunity through the secretion of extracellular vesicles (EVs) containing microRNAs (miRNAs).

**Methods:**

In this study, hydatid antigens were isolated from sheep livers and mice peritoneal cavities. MSCs derived from mouse bone marrow were treated with different hydatid antigens, and EVs were isolated and characterized from the conditioned medium of MSCs. Small RNA library construction, miRNA target prediction, and differential expression analysis were conducted to identify differentially expressed miRNAs. Functional enrichment and network construction were performed to explore the biological functions of the target genes. Real-time PCR and Western blotting were used for miRNA and gene expression verification, while ELISA assays quantified TNF, IL-1, IL-6, IL-4, and IL-10 levels in cell supernatants.

**Results:**

The study successfully isolated hydatid antigens and characterized MSC-derived EVs, demonstrating the impact of antigen concentration on MSC viability. Key differentially expressed miRNAs, such as miR-146a and miR-9-5p, were identified, with functional analyses revealing significant pathways like Endocytosis and MAPK signaling associated with these miRNAs’ target genes. The miRNA-HUB gene regulatory network identified crucial miRNAs and HUB genes, such as Traf1 and Tnf, indicating roles in immune modulation and osteogenic differentiation. Protein–protein interaction (PPI) network analysis highlighted central HUB genes like Akt1 and Bcl2. ALP activity assays confirmed the influence of antigens on osteogenic differentiation, with reduced ALP activity observed. Expression analysis validated altered miRNA and chemokine expression post-antigen stimulation, with ELISA analysis showing a significant reduction in CXCL1 expression in response to antigen exposure.

**Conclusion:**

This study provides insights into the role of MSC-derived EVs in regulating parasite immunity. The findings suggest that hydatid antigens can modulate the expression of miRNAs in MSC-derived EVs, leading to changes in chemokine expression and osteogenic capacity. These findings contribute to a better understanding of the immunomodulatory mechanisms involved in hydatid disease and provide potential therapeutic targets for the development of new treatment strategies.

## Background

Parasitic infections continue to pose a significant health challenge in developing countries worldwide, affecting humans, domesticated animals, and livestock. Hydatid disease, also known as cystic echinococcosis, is a zoonotic infection caused by *Echinococcus* parasites, particularly *Echinococcus granulosus* (Gottstein et al., 2014). The disease occurs when the oncosphere within the parasite’s eggs hatches after accidental ingestion by an intermediate host, such as herbivores or humans. The oncosphere then penetrates the intestinal wall, enters the host’s systemic circulation via the portal vein, and eventually colonizes a terminal blood vessel (Brunetti et al., 2010). Hydatid disease has a gradual onset and can affect various tissues and organs in the body, causing significant health damage. The treatment of this disease is often complicated by the spread of the lesions (Agudelo et al., 2016). Sheep, as natural intermediate hosts, are common donors for obtaining fertile cysts, and mice, as common model organisms, are also frequently used in the study of the mechanisms of echinococcosis.

Upon observing hydatid lesions, it is evident that cystic hydatid disease is characterized by the presence of hydatid cysts. These cysts contain specific antigen types, including excretory-secretory products (ESPs) secreted by the protoscolex within the inner cysts, the hydatid cyst fluid (HCF) that fills the inner cysts, and the laminated layer particles (pLL) formed by the shedding of the laminated layer of the cysts. All three of these antigens have been found to interact with the host’s internal environment and immune system (Wen et al., 2019). Mesenchymal stem cells (MSCs) are pluripotent stem cells that are distributed throughout the body and have the potential to differentiate into various tissue types. They are also believed to have the ability to regulate innate and adaptive immunity, partly through the secretion of extracellular vesicles (EVs) (Zhuang et al., 2021).

In recent years, the field of extracellular vesicle (EV) research has emerged as a frontier in the quest for novel diagnostic and therapeutic tools for a range of diseases, including infectious diseases and cancer (Harvey et al., 2022; Schweer et al., 2022). EVs are vesicles surrounded by a lipid membrane that are released by cells into the extracellular compartment. They are secreted by almost all cell types under normal physiological conditions, and their composition varies depending on the cell type and physiological state. Importantly, the regulatory functions of EVs are known to be altered during parasitic infections, highlighting their potential as innovative therapeutic agents and vaccine targets (Keshtkar et al., 2022). Previous studies have shown that EVs derived from MSCs contain non-coding RNAs, particularly microRNAs (miRNAs), which can be transferred to recipient cells and play regulatory roles (Cai et al., 2020; Mazziotta et al., 2023b). miRNAs are small, endogenous RNAs approximately 20–24 nucleotides long that play a role in post-transcriptional gene expression regulation in both plants and animals (Saliminejad et al., 2019; Li et al., 2021).

Based on the distribution of MSCs around vascular tissues in the body (Murray et al., 2014), it is hypothesized that they are one of the first cell types to come into contact with hydatid lesions. Previous studies have demonstrated that MSCs can induce changes in chemokine expression and osteogenic activity in themselves and surrounding cells through EVs (Dabrowska et al., 2021). Chemokine expression plays a crucial role in parasite immunity by recruiting multiple immune cells and is one of the main immunomodulatory mechanisms of MSCs (Yao et al., 2021; Zhang et al., 2023). Furthermore, *in vivo* experiments have revealed that transplantation of MSCs can improve the prognosis of hydatid disease by mitigating tissue damage and modulating the immune response, underscoring the significance of MSCs in the parasite–host interaction (Kian et al., 2022). Osteogenic differentiation is an important pathway involved in maintaining physiological bone homeostasis in bone marrow MSCs. In this study, we are interested in exploring the miRNA expression profiles within EVs derived from MSCs after stimulation with different antigens. We also aim to investigate the changes in chemokine expression and osteogenic capacity of these EVs. By comparing the changes in miRNA expression profiles after antigen stimulation, we hope to gain further insights into the role of MSC-derived EVs in regulating parasite immunity.

## Materials and methods

### Collection of hydatid antigens

**Figure 1:**
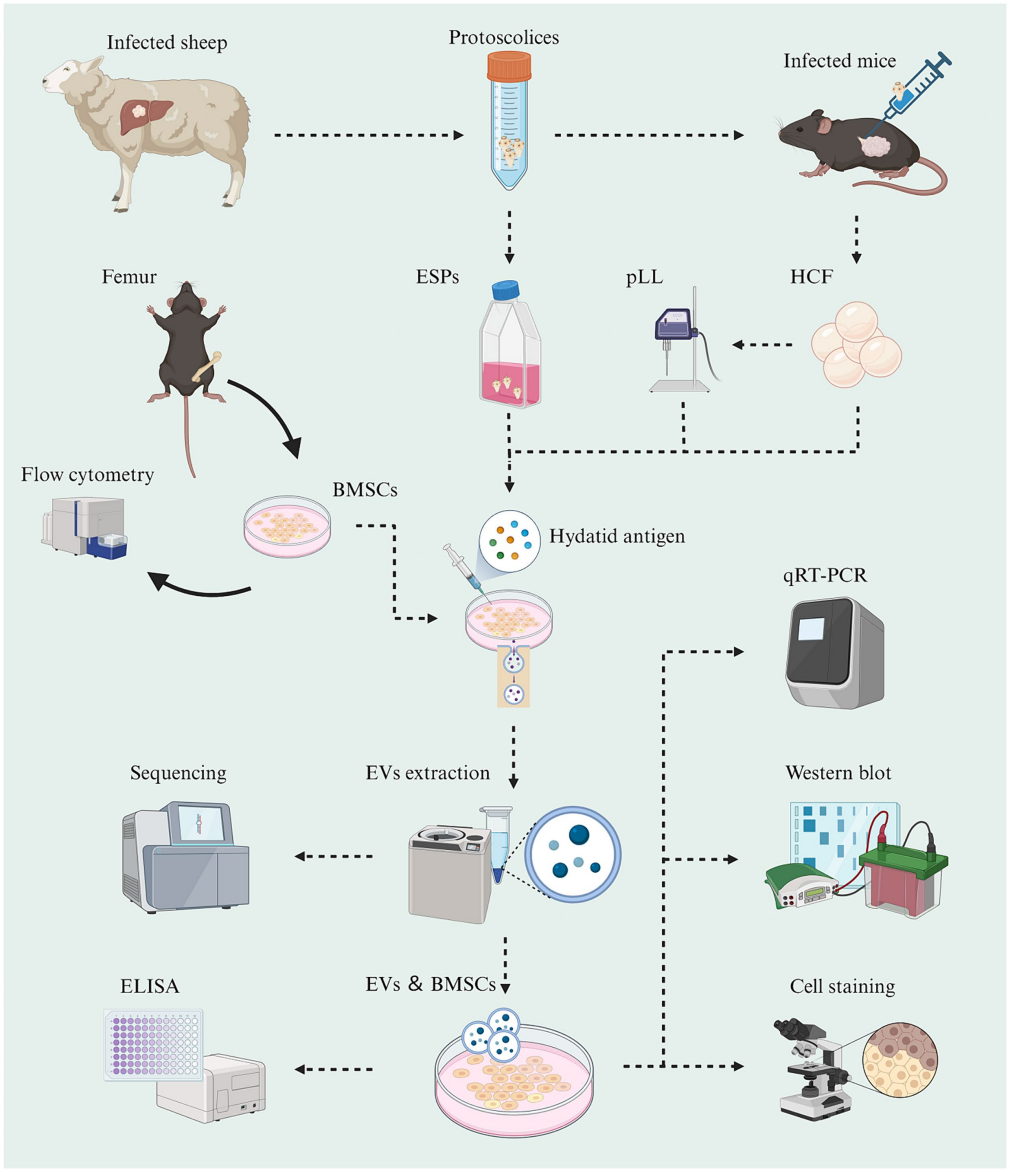
Schematic graph showing the experimental workflow of this study (Created with BioRender.com).

Livers of sheep infected with Echinococcus granulosus were obtained from the slaughterhouse. Cysts containing Protoscolex were extracted by puncturing the active cysts using a sterile syringe in the laboratory aseptic bench. Protoscolex were collected by centrifuging the cysts at 1000 × g for 10 min and washed with sterile PBS three times. The Protoscolex were then added to α-MEM medium at a density of 7,500/ml and transferred to aseptic cell culture flasks. The medium containing ESPs was obtained by centrifugation after 24 h of incubation at 37°C under 5% CO_2_. Some of the extracted Protoscolex were injected into the abdominal cavity and paraspinal column of mice at a concentration of 2000/100 μl. After 6 months, the fluid from the cysts was extracted by dissecting and exposing the cysts and centrifuged at 1,000 × *g* for 10 min to collect the supernatant (Figure 1). The α-MEM medium enriched with ESPs or pLL, as well as HCF, was filtered through a 0.22 μm filter, supplemented with 5% exosome-free fetal bovine serum (Vivacell, Shanghai, China) and penicillin–streptomycin, and stored at −80°C until further use. Protein quantification of the hydatid antigens was performed using the BCA kit.

### Isolation and characterization of MSCs

6-8-week-old SPF-grade C57BL/6 mice were purchased from the Experimental Animal Center of Xinjiang Medical University. The femur and tibia were removed from the aseptic table, and the medullary cavity was exposed. The marrow cavity was blown and rinsed using cell culture medium. The rinsed bone marrow tissues were collected, and the bone marrow cells were collected by centrifugation after filtration through a 70-μm sieve. The cells were resuspended in α-MEM medium containing 10% fetal bovine serum and penicillin–streptomycin and then spread evenly on the cell culture dish. The dish was placed in a 37°C, 5% CO_2_ incubator, and the cell culture medium was changed every 2 days. When the monolayer of cells was spread over the bottom of the dish, they were digested with trypsin and passaged in a 1:2 ratio. The 3rd generation cells were used for flow cytometric identification and subsequent experiments.

Flow cytometry was employed to characterize the isolated MSCs. The cultured MSCs up to the third generation were washed, digested, and centrifuged to prepare a single-cell suspension. Specific fluorescent-labeled flow cytometry antibodies targeting positive markers (CD29, CD90) on the cell surface were used for detection, while negative markers (CD45, CD11b/c) served as controls to exclude potential cell contamination. The flow cytometer settings were adjusted to appropriate excitation wavelengths and fluorescence channels for the detection and quantification of cell surface markers.

### Cell proliferation and activity assay

To assess the effect of hydatid antigens on MSC survival and proliferation, MTT assays were performed according to the manufacturer’s instructions (Qu et al., 2020). BMSCs were seeded in 96-well plates at a density of 10^4^ cells/well and cultured overnight. ESPs were added to the wells at concentrations ranging from 0 to 2 μg/ml, HCF at concentrations ranging from 0 to 180 μg/ml, and pLL at concentrations ranging from 0 to 60 μg/ml. After incubation for 24–48 h, MTT solution was added to each well and incubated for an additional 4 h. The absorbance was measured at 490 nm using an enzyme labeling detector. Each experiment was performed with 3 replicates.

### Cell treatment

BMSCs were cultured in α-MEM medium supplemented with 10% exosome-free fetal bovine serum. When the cell confluence reached 70%, ESPs, HCF, and pLL were added to the medium of each group at a concentration of 2 μg/mL, achieved through a dilution of 1:5 for ESPs, 1:450 for HCF, and 1:150 for pLL. The petri dishes were then incubated in a 37°C, 5% CO2 atmosphere for 4 days, with the complete culture medium refreshed every 2 days. Subsequently, the supernatant of the incubated medium was collected, centrifuged, and stored at −80°C for further use (Levy et al., 2023). Each experimental group was performed in triplicate to ensure reproducibility and accuracy.

### Extraction and characterization of EVs

Supernatants the cell cultures were thawed at 4°C, transferred to new centrifuge tubes, and centrifuged at 10,000 × g for 45 min at 4°C to remove larger vesicles. The supernatant was then filtered through a 0.45 μm filter membrane and centrifuged at 100,000 × g for 70 min at 4°C. After resuspension with 10 mL of pre-cooled 1 × PBS, the supernatant was again ultracentrifuged at 100,000 × g for 70 min at 4°C. The supernatant was removed and resuspended with 100 μL of pre-cooled 1 × PBS. Transmission electron microscope (TEM) identification, particle size analysis, and protein extraction were performed. The remaining EVs were lysed with TRIzol, and total RNA was extracted for sequencing and qRT-PCR identification. Protein concentrations of BMSC-EVs were measured using the BCA Protein Concentration Assay Kit.

### Small RNA library construction and data quality control

Total RNA was extracted from EVs using the Trizol method. The concentration and quality of RNA were determined using a spectrophotometer and agarose gel electrophoresis. Small RNA-seq libraries were constructed using the NEBNext Small RNA Library Prep Kit and sequenced on the Illumina NovaSeq platform. Quality control of the sequences was performed using Fastp software. The sequences were compared with the mature miRNA sequences of mice in the miRBase database^1^ and Rfam database,^2^ and the successfully matched miRNAs were used for subsequent analysis.

### Target gene prediction and differential analysis of miRNAs

The miRDeep2 software^3^ was used to compare the small RNA sequences with the miRNA precursors and mature bodies of the corresponding species in the miRBase database. The expression of known miRNAs was counted, and significantly differentially expressed miRNAs were identified based on adjusted *p*-value and fold change. Target genes of the RNAs were predicted using miRanda^4^ and RNAhybrid software,^5^ and the overlapping predictions were considered as the final result. The miRNAs and target genes were grouped based on their expression levels relative to the control group.

### GO and KEGG enrichment analysis of target genes

The target genes of differentially expressed miRNAs were subjected to GO and KEGG enrichment analysis using the Metascape website.^6^ The parameters for the enrichment analysis were set according to the specified criteria (Mazziotta et al., 2023a).

### Construction of protein–protein interaction networks

The target genes of up- and down-regulated miRNAs were uploaded to the STRING database^7^ for the construction and visualization of PPI networks.

### Construction of transcription factor-miRNA networks

The target genes analyzed by enrichment in the KEGG analysis were uploaded to the NetworkAnalyst 3.0 website.^8^ Transcription factor (TF) and gene target data derived from the ENCODE ChIP-seq data were used. The TF gene regulatory networks and miRNA-target gene networks were imported into Cytoscape software^9^ for visualization.

### Preparation of ESP-EVs and cellular intervention

BMSCs were cultured in α-MEM medium containing 10% exosome-free FBS and penicillin–streptomycin. After reaching 70% confluence, 2 μg/ml of ESPs were added to the culture for 48 h. EVs were extracted from the cell supernatants, resuspended in culture medium, and labeled as ESP-EVs. BMSCs were seeded in 6-well plates at a density of 2 × 10^5^ cells per well. 2 μg/ml of ESPs and ESP-EVs were added to the corresponding groups, and the control group was added with an equal volume of complete medium. The cells were cultured for 10 days with the medium changed every 2 days. Total proteins and total RNA samples were extracted from the cells for further analysis.

### Alkaline phosphatase staining

BMSCs were seeded in 24-well plates at a density of 1 × 10^4^ cells per well. After attachment, the medium was replaced with osteogenic induction medium. 2 μg/ml of ESPs and ESP-EVs were added to the corresponding groups. The cells were cultured for 10 days with the medium changed every 2 days. Alkaline phosphatase (ALP) staining was performed, and the stained cells were photographed under an inverted microscope. Three replicates were set up for each stained well.

### Real-time PCR validation

Total RNA was extracted using the Trizol method, and cDNAs were synthesized using a reverse transcription kit. Real-time PCR was performed using a real-time fluorescent quantitative PCR system, and the relative expression of target genes was calculated using the ΔCt method. GAPDH and U6 were used as internal reference genes. The primer sequences used for amplification are shown in Table 1. Each experiment included three technical replicates.

**Table 1:** Primer sequences used in qRT-PCR.

Genes	Forward (5′-3′)	Reverse (5′-3′)/RT Primer
Cxcl1	TGGCTGGGATTCACCTCAAGAAC	GTGTGGCTATGACTTCGGTTTGG
Cxcl5	CTTCCTCAGTCATAGCCGCAACG	TGACTTCCACCGTAGGGCACTG
Alpl	TCATTCCCACGTTTTCACATTC	GTTGTTGTGAGCGTAATCTACC
mmu-miR-9-5p	GAGCGCGTCTTTGGTTATCTAGC	GTCGTATCCAGTGCAGGGTCCGAGGTATTCGCACTGGATACGACTCATAC
mmu-miR-146a-5p	CGCGTGAGAACTGAATTCCA	GTCGTATCCAGTGCAGGGTCCGAGGTATTCGCACTGGATACGACAACCCA

### Western blot assay

Proteins were extracted from EVs and BMSCs, separated by SDS-PAGE, and transferred to PVDF membrane. The membrane was incubated with primary antibodies and then with HRP-labeled secondary antibodies. Immunoreactivity was detected using chemiluminescent substrate, and the bands were analyzed for grayscale values. Each experiment was performed with 3 replicates.

### Enzyme linked immunosorbent assay

ELISA was performed to measure TNF, IL-1, CXCL1, IL-4, and IL-10 levels in cell supernatants collected from MSCs cultured under the same conditions as the sequencing analysis. Samples were incubated with the ELISA plate at 37°C, followed by the addition of biotinylated antibody working solution and enzyme conjugate working solution. Substrate (TMB) was added, and the OD values were measured at 450 nm after incubation. The target concentration was calculated as the difference between day 4 and day 0. Each experiment included three technical replicates.

### Analytical approaches

All bioinformatics analyses were conducted by two trained independent operators at separate time points, and the results were compared to validate the consistency and reliability of the findings. The data in this study were analyzed and visualized using GraphPad Prism software (version 8.0). Measurement data were presented as mean ± standard deviation. An independent sample *t*-test was employed for comparing two groups, while one-way analysis of variance was used for comparing multiple groups. Statistical significance was denoted as * (*p* < 0.05), ** (*p* < 0.01), *** (*p* < 0.001), and **** (*p* < 0.0001).

## Results

### Characterization of hydatid antigens and MSC-derived EVs

**Figure 2:**
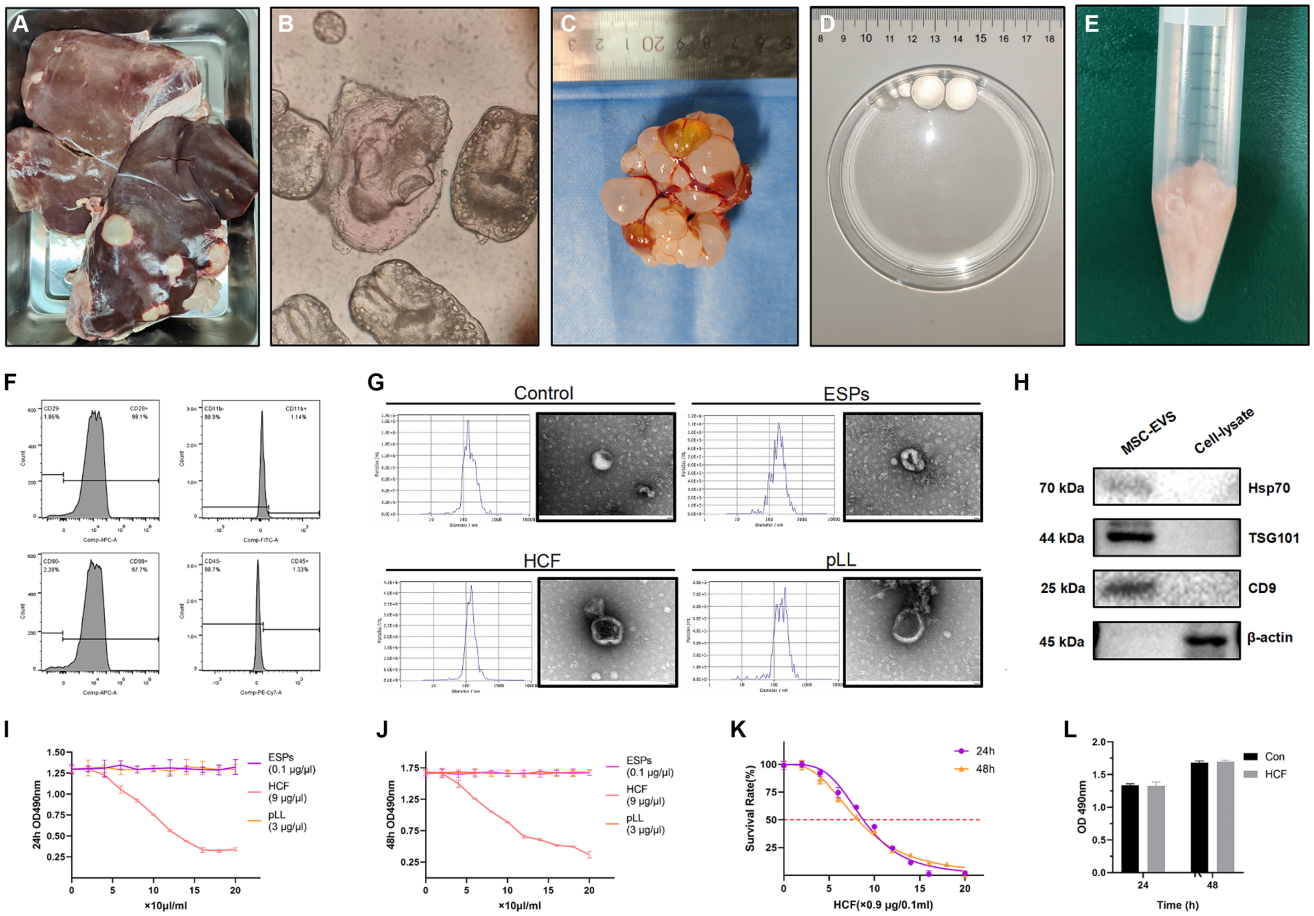
**(A)** Liver of a sheep infected with hydatid disease, used for the extraction of Protoscolex. **(B)** Protoscolex of *Echinococcus granulosus* observed under the microscope. **(C)** Hydatid cysts extracted from the peritoneal cavity of mice for HCF and pLL analysis. **(D)** Isolated daughter cysts were utilized for HCF and pLL extraction. **(E)** The inner cysts, post cyst fluid extraction, were employed for pLL extraction. **(F)** Flow cytometry was used to identify the characteristic positive surface markers (CD29, CD90) and negative markers (CD45, CD11b/c) of MSCs. **(G)** The size and morphology of EVs were assessed using NTA and TEM. **(H)** Western blot analysis was conducted to detect the expression of characteristic EV proteins (Hsp70, TSG101, CD9), using lysates from the same batch of cells and intracellular structural proteins (β-actin) as controls. **(I,J)** MTT assay analysis of cell viability after treating BMSCs with three different antigens (ESPs, HCF, pLL) at varying concentrations for 24 and 48 h. **(K)** MTT assay analysis of cell survival rate after treatment with different concentrations of HCF for 24–48 h. **(L)** MTT assay analysis of cell viability at 24 and 48 h after adding 7.2 μg/ml HCF, compared to the control group. In the MTT assay, a higher absorbance at 490 nm (OD 490) indicates greater cell viability. Values in the images are represented as mean ± standard error of the mean (SEM). Differences in means and SEMs across three independent experiments were evaluated using Student’s *t*-test. *p < 0.05, **p < 0.01, ***p < 0.001 compared to the control group.

Protoscoleces, ESPs, HCF, and pLL were successfully extracted from sheep livers and mice peritoneal cavities post-infection (Figures 2A–E). The flow cytometry analysis of BMSCs from C57BL/6 mice revealed significant expression of CD29 and CD90 markers, while CD45 and CD11b were not prominent (Figure 2F). EVs isolated from cultured supernatants conformed to the ISEV standards, with particle sizes ranging from 30 to 800 nm, and an average size for each group (Control: 170.4 nm; ESPs: 205.5 nm; HCF: 151.3 nm; pLL: 182.5 nm). TEM confirmed the presence of vesicles with the characteristic cup-shaped morphology, typically measuring between 100 and 150 nm (Figure 2G). The western blot analysis verified the expression of exosomal markers HSP70, TSG101, and CD9 in the EV preparations, and the absence of the intracellular protein, β-actin (Figure 2H). Protein quantification using the BCA assay indicated that Protoscoleces secreted approximately 10 μg/ml of protein over 24 h, while the average protein concentrations for mouse-derived HCF and pLL were 900 μg/ml and 300 μg/ml, respectively.

### MTT assay for antigenic concentration determination

The MTT assay was utilized to determine the impact of varying antigen concentrations on BMSC proliferation. The results indicated that ESPs and pLL at concentrations ranging from 0 to 2 μg/ml and 0 to 60 μg/mL, respectively, did not significantly affect cell proliferation after 48 h in comparison to the control group. For HCF, semi-inhibitory concentrations (IC50) of 78.9 and 73.3 μg/ml were determined at 24 and 48 h, respectively. A concentration of 7.2 μg/ml of HCF did not significantly impact BMSC survival (Figures 2I–L).

### Classification of small RNAs in EVs

EVs contain various types of small RNAs, including miRNAs, rRNAs, tRNAs, and snRNAs. Through database comparison, it was found that the control, ESPs, HCF, and pLL groups contained an average of 0.33, 0.6, 0.43, and 0.63% of known mature miRNAs, respectively. For rRNA, the percentages were 3.4, 5.2, 3.43, and 4.56% in the control, ESPs, HCF, and pLL groups, respectively. As for snRNA, the percentages were 0.16, 1.83, 0.13, and 0.2% in the respective groups, while for tRNA, the percentages were 3.46, 1.83, 3.26, and 3.53% in the control, ESPs, HCF, and pLL groups, respectively.

### miRNA expression analysis

**Figure 3:**
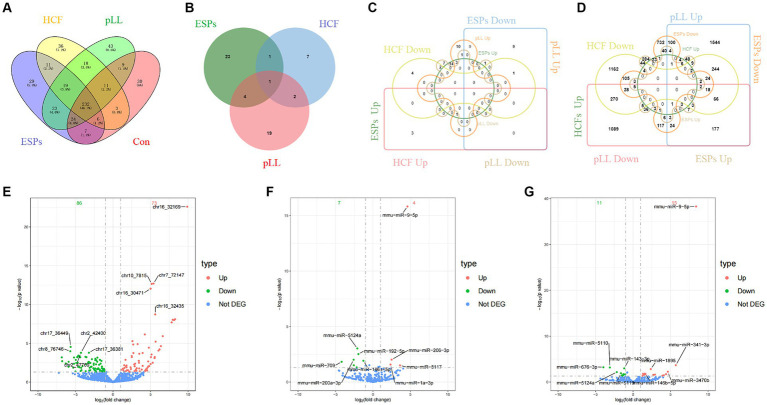
**(A)** The number and overlap of mature miRNAs identified within EVs of the control group and the three antigen-treated groups (ESPs, HCF, pLL). **(B)** Differential miRNA number and overlap of the three antigen-treated groups compared to the control group. **(C)** The number and overlap of miRNAs in each of the three antigen-treated groups compared to the control group after the differential miRNAs continued to be grouped according to up-/down-regulated expression. **(D)** The number and overlap of miRNA target genes in each of the three antigen-treated and control groups after the differential miRNAs continued to be grouped according to up-/down-regulated expression. **(E)** Volcano plot of differentially expressed miRNAs in the ESPs group. **(F)** Volcano plot of differentially expressed miRNAs in the HCF group. **(G)** Volcano plot of differentially expressed miRNAs in the pLL group.

A total of 322, 351, 336, and 379 known mature miRNAs were identified within the EVs of the control, ESPs, HCF, and pLL groups, respectively. Notably, 29 miRNAs were differentially expressed in the ESPs group compared to the control group, with 18 up-regulated and 11 down-regulated, impacting 1729 target genes. The HCF group exhibited 11 differentially expressed miRNAs, with 4 up-regulated and 7 down-regulated, linked to 2045 target genes. In the pLL group, 26 miRNAs showed differential expression, with 15 up-regulated and 11 down-regulated, corresponding to 4,156 target genes (Figures 3A–G and Supplementary Figure S1).

### GO and KEGG enrichment analysis of target genes

GO enrichment analysis revealed that target genes were predominantly enriched in biological processes such as exocytosis, cellular response to oxidative stress, and Ras protein signal transduction. In terms of cellular components, enrichment was observed in the cytoplasmic region and actin cytoskeleton. Molecular functions such as small GTPase binding and protein serine/threonine kinase activity were also highlighted (Supplementary Figure S2).

The KEGG enrichment analysis provided further insights, showing that target genes were primarily involved in signaling pathways such as Endocytosis, MAPK, Ras, and cAMP, as well as in the regulation of stem cell pluripotency (Supplementary Figure S3).

### miRNA-HUB gene regulatory network analysis

Through the TRRUST database, transcription factor genes and their regulatory relationships were identified in six miRNA-groupings: ESPs-Up/Down, HCF-Up/Down, and pLL-Up/Down. Key regulatory miRNAs, including miR-9-5p, miR-124, and miR-23a-3p, and HUB genes such as Traf1 and Tnf, were recognized within the constructed TF-miRNA networks using NetworkAnalyst3.0 and Cytoscape software (Supplementary Figure S5). Detailed information on the TF genes and their regulatory targets is presented in Tables 2, 3.

**Table 2:** TF genes and their overlapping genes in the target genes of upregulating miRNA.

Group	Key TF	overlapped genes	*p* value	List of overlapped genes
ESP/ Control	Foxo1	2	0.006	Akt1,Ccr7
pll/ Control	Sp1	11	0.000	Ngfr,Egfr,Il5ra,Oprm1,Bmp7,Csf1,Vegfa,Ets1,Oprd1,Tgfbr2,Insr
	Nfkb1	8	0.002	Shh,Slc1a2,Xiap,Csf1,Egfr,Itgam,Vegfa,Bcl2
	Rela	7	0.001	Xiap,Cacna1c,Gabre,Slc1a2,Csf1,Shh,Bcl2
	Egr1	6	0.000	Wnt4,Mapk8,Tgfbr2,Ngfr,Fgf2,Igf1r
	Esr2	5	0.000	Vegfa,Igf1r,Casp8,Bcl2,Mapk8
	Ets1	5	0.001	Vegfa,Tgfbr2,Oprd1,Angpt2,Cd4
	Sp3	5	0.003	Oprd1,Oprm1,Csf1,Vegfa,Angpt2
	Trp53	5	0.045	Casp8,Bcl2,Gabre,Igf1r,Insr
	Wt1	4	0.000	Vegfa,Csf1,Wnt4,Igf2
	Nrf1	4	0.000	Sirt6,Grin1,Grin2b,Nos1

**Table 3:** TF genes and their overlapping genes in the target genes of downregulating miRNA.

Group	Key TF	overlapped genes	*p* value	List of overlapped genes
ESP/ Control	Sp1	15	0.000	Mmp2,Cldn19,Pld2,Slc2a1,Csf1,Dnm1,Cebpa,Tnf,Cdkn1a,Parp1,Wnt9a,Jund,Itga5,Ghr,Thra
	Trp53	12	0.000	Foxp3,Gadd45a,Ei24,Runx1,Foxo3,Cdkn1a,Rela,Pdgfrb,Mmp2,Perp,Sin3a,Ddit4
	Nfkb1	12	0.000	Cxcl10,Mmp2,Bcl2l1,Cebpa,Cdkn1a,Foxp3,Slc2a1,Csf1,Ctnnb1,Tnf,Ebi3,Mapk14
	Rela	7	0.000	Ebi3,Bcl2l1,Cdkn2a,Cxcl10,Csf1,Cldn5,Tnf
	Irf1	5	0.000	Cdkn1a,Cxcl10,Mapk14,Foxp3,Tnf
	Ets1	5	0.000	Il5,Itga2b,Foxp3,Cdkn2a,Cd4
	Egr1	5	0.000	Mapk14,Rcan1,Tnf,Gadd45a,Jund
	Stat3	5	0.000	Mmp2,Stat3,Bcl2l1,Rela,Tnf
	Sp3	5	0.001	Cdkn1a,Dnm1,Ghr,Csf1,Cebpa
	Jun	5	0.004	Slc2a1,Itga5,Mmp2,Cdkn2a,Tnf
HCF/ Control	Sp1	8	0.000	Dck,Src,Mapk1,Cdk6,Slc3a2,Dnm1,Sh3gl1,Tnf
	Trp53	6	0.000	Pml,Src,Pdgfrb,Cdkn1b,Igf1r,Mapk1
	Stat3	4	0.001	Nos2,Cdkn1b,Tnf,Cav1
	Jun	4	0.006	Src,Eno2,Nos2,Tnf
	Twist1	3	0.001	Fgfr2,Tnf,Il6ra
	Myc	3	0.003	Pdgfrb,Cav1,Eif4e
	Ctnnb1	3	0.005	Pml,Mitf,Wnt1
	Sp3	3	0.011	Dnm1,Mapk1,Dck
	Rela	3	0.031	Cdkn1b,Nos2,Tnf
	Tfap2c	2	0.002	Fgf4,Cdk6
pll/ Control	Sp1	11	0.001	Lmna,Mmp2,Fos,Plaur,Mapk1,Csf1r,Tnf,Alox5,Jak3,Jund,Thra
	Nfkb1	9	0.001	Mmp2,Il2ra,Nos2,Alox5,Relb,Fos,Tnf,Plaur,Mapk14
	Jun	8	0.000	Plaur,Relb,Fos,Mmp2,Nos2,Smurf1,Tnf,Csf1r
	Stat3	6	0.001	Mmp2,Nos2,Jak3,Twist2,Fos,Tnf
	Ep300	5	0.001	Axin2,Fos,Gata4,Nos2,Mmp2
	Egr1	5	0.002	Mapk14,Tnf,Ache,Lhb,Jund
	Rela	5	0.021	Ptpn1,Fos,Cacna1c,Nos2,Tnf
	Sox10	5	0.000	Sufu,L1cam,Dhh,Ret
	Spi1	4	0.002	Csf1r,Tnf,Itgb3,Fes
	Ctnnb1	4	0.008	Lhb,Ephb2,Axin2,Wnt1

### PPI network construction

PPI networks were constructed for each miRNA-grouping related to KEGG pathway enrichment. The networks varied in complexity, with the ESPs-Up group having a network of 50 nodes and 69 edges, while the pLL-Down group had a network with 267 nodes and 1,212 edges (Supplementary Figure S4). Central HUB genes, including Akt1, Tnf, and Bcl2, were identified based on node connectivity within these networks (Table 4).

**Table 4:** The top 5 HUB genes in each group of PPI networks according to the degree value.

Group	Gene	Description	Node degree
ESPs up	Akt1	RAC-alpha serine/threonine-protein kinase	20
	Gnb3	Guanine nucleotide-binding protein G(I)/G(S)/G(T) subunit beta-3	9
	Rras	Ras-related protein R-Ras	7
	Adcy6	Adenylate cyclase type 6	6
	Calm5	Skin calmodulin-related protein 2	6
ESPs down	Ctnnb1	Catenin beta-1	57
	Tnf	Tumor necrosis factor	55
	Stat3	Signal transducer and activator of transcription 3	54
	Mapk14	Mitogen-activated protein kinase 14	44
	Cd4	T-cell surface glycoprotein CD4	37
HCF up	Epha1	Ephrin type-A receptor 1	6
	Epha2	Ephrin type-A receptor 2	6
	Efna2	Ephrin-A2	5
	Epha4	Ephrin type-A receptor 4	5
	Rasa1	RAS p21 protein activator 1	5
HCF down	Src	Neuronal proto-oncogene tyrosine-protein kinase Src	32
	Tnf	Tumor necrosis factor, membrane form	27
	Pik3ca	Phosphatidylinositol 4,5-bisphosphate 3-kinase catalytic subunit alpha isoform	26
	Igf1r	Insulin-like growth factor 1 receptor alpha chain	21
	Nras	GTPase NRas	21
pll up	Kras	GTPase KRas, N-terminally processed	75
	Egfr	Epidermal growth factor receptor	61
	Bcl2	Apoptosis regulator Bcl-2	51
	Gnao1	Guanine nucleotide-binding protein G(o) subunit alpha	45
	Dlg4	Disks large homolog 4	43
pll down	Tnf	Tumor necrosis factor, membrane form	60
	Grb2	Growth factor receptor-bound protein 2	53
	Mapk1	Mitogen-activated protein kinase 1	46
	Mapk14	Mitogen-activated protein kinase 14	46
	Calm3	Calmodulin-1	45

### Cellular ALP activity assay

**Figure 4:**
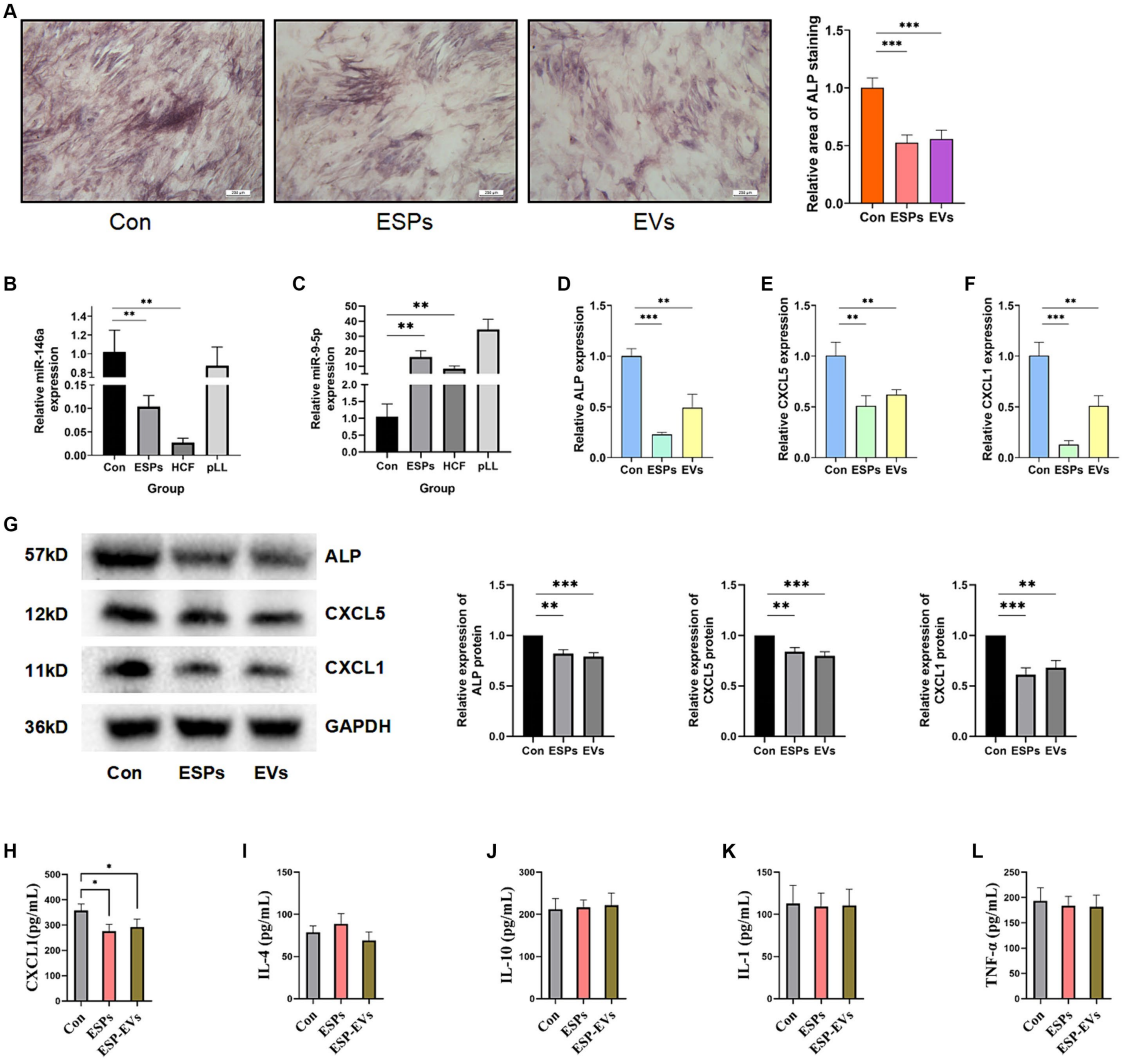
**(A)** ALP staining of BMSCs and area statistics after 10 days of osteogenic induction in control, ESPs, and ESP-EVs groups. **(B)** Relative expression of miRNA-146a in the three groups. **(C)** Relative expression of miRNA-9-5p in the three groups. **(D)** ALP mRNA expression in BMSCs after 10 days of ESPs and ESP-EVs intervention. **(E)** CXCL5 mRNA expression in BMSCs after 10 days of ESPs and ESP-EVs intervention. **(F)** CXCL1 mRNA expression in BMSCs after 10 days of ESPs and ESP-EVs intervention. **(G)** Statistics of ALP, CXCL5, CXCL1 protein expression in BMSCs after 10 days of ESPs and ESP-EVs intervention. **(H-I)** The ELISA experiment results show the concentrations of CXCL1, IL-4, IL-10, IL-1, and TNF-α in the cell supernatant. Values in the images are represented as mean ± standard error of the mean (SEM). Differences in means and SEMs across three independent experiments were evaluated using Student’s *t*-test. *p < 0.05, **p < 0.01, ***p < 0.001 compared to the control group.

Osteogenic induction was assessed by measuring ALP activity in BMSCs after treatment with ESPs and ESP-EVs. Both treatments significantly reduced ALP activity compared to the control group, indicating potential effects on osteogenic differentiation (Figure 4A).

### Validation of miRNA and gene expression

The expression levels of miR-146a and miR-9-5p, which were randomly selected from all differentially expressed miRNAs for validation, were validated using qRT-PCR. The expression of miR-146a was found to be significantly lower in the ESPs and HCF groups, while the expression of miR-9-5p was significantly higher in the ESPs, HCF, and pLL groups compared to the control group (Figures 4B,C). Consistent with these findings, the mRNA and protein expression levels of CXCL1 and CXCL5, as well as ALP, were significantly lower in the ESPs and ESP-EVs groups compared to the control group (Figures 4D–F,G).

### ELISA analysis of cytokine expression in cell supernatants

ELISA analysis of the experimental groups exposed to antigen ESPs and ESP-EVs demonstrated a significant reduction in the expression of CXCL1 compared to the control group. However, no significant alterations were observed in the expression of TNF-α, IL-1, IL-4, and IL-10 among the three groups (Figures 4H–L).

## Discussion

Cystic echinococcosis, a zoonotic infection common in livestock areas, is caused by adult worms residing in the intestines of dogs and other canids. Eggs excreted by these worms in feces contaminate the environment. When hoofed animals like goats, sheep, and pigs ingest the contaminated eggs, the oncospheres released from the eggs travel through the bloodstream and parasitize various organs. Humans can also be infected as occasional intermediate hosts.

The most common sites of occurrence of hydatid cysts in humans are the liver and lungs, but involvement of other parts of the body, such as the kidneys, spleen, and skeleton, has also been reported. The characteristic hydatid lesions consist of a fibrous outer capsule formed by host reactants, except in skeletal lesions, and an inner capsule that contains an outer acellular layer (laminated layer) and a medial germinative layer. These lesions are filled with clear cystic fluid (Gessese, 2020). Of particular interest in our study is the pathologic process of skeletal hydatids. Due to the slow progression of the disease and the lack of specific manifestations in the early stages, patients with skeletal hydatids often present to the clinic with localized masses or pathologic fractures after a long incubation period of several years to more than a decade. The disruption of the patient’s ability to work is often catastrophic. Surgical removal of hydatid lesions within the bone marrow cavity is difficult due to the widespread dissemination of the lesions and the ineffectiveness of chemotherapy. Similar challenges are faced in the resection of tumor tissue (Song et al., 2007).

In addition to traditional surgical and chemotherapeutic approaches, various treatment options are being explored for hydatid disease. One such approach is the transplantation of mesenchymal stem cells (MSCs), which has been used in the treatment of cardiovascular and degenerative diseases. It has also been reported to influence prognosis and inflammatory status in *in vivo* experiments. However, the use of MSCs transplantation does not differentiate between antigen types and does not provide additional reference for further mechanistic exploration.

The complex parasitic cycle of *Echinococcus* parasites is believed to involve certain immune evasion mechanisms that allow the parasites to modulate and reduce the immune response of the host. Antigens from these parasites have been shown to modulate the activity, differentiation, and polarization of immune cells such as dendritic cells and macrophages (Chulanetra and Chaicumpa, 2021). Bone tissue, which is rich in MSCs and immune cells, may be susceptible to the regulatory effects of hydatid antigens.

MSCs are pluripotent stem cells found in diverse tissue types. In addition to their capacity for differentiation into osteoblasts and chondrocytes (Kim et al., 2022), MSCs within bone tissue serve as integral components of the bone immune microenvironment, exerting a crucial role in immune regulation (Anand et al., 2023). These properties have made MSCs a focus of therapeutic potential. Paracrine secretion is the main mechanism through which MSCs exert their immunomodulatory effects, and extracellular vesicles (EVs) released by MSCs play a vital role in intercellular communication. EVs contain a diverse array of proteins, lipids, and nucleic acids, with miRNAs being the main type of RNA involved in the immunoregulatory effects of MSC-derived EVs.

In our study, we observed that different hydatid disease antigenic stimuli were able to alter the intravesicular miRNA expression profiles of MSCs. The differences in expression profiles caused by different antigenic stimuli were relatively obvious, indicating that MSCs are capable of sensing and responding to different antigenic types in hydatid disease. Previous studies have shown that various miRNAs are involved in the immunoregulation of MSCs. Therefore, the functional analysis of differentially expressed miRNAs can help in understanding the role of MSCs in parasite immunity.

Among the differentially expressed miRNAs, miR-9-5p showed significant changes in all three groups relative to the control group. This suggests that miR-9-5p may have a representative role in the immunomodulation of hydatid disease. Previous studies have shown that miR-9-5p can promote the anti-inflammatory effects of MSCs, inhibit osteogenic differentiation and mineralization of osteoblast lineage cells, and promote osteoclastogenesis through the activation of the SIRT1/NFκB signaling pathway (Zhang L. et al., 2021; Gao et al., 2022; He et al., 2022). This indicates that miR-9-5p has the potential to modulate immunosuppression and regulate the osteogenic/osteoclastic balance in hydatid disease.

Interestingly, miR-146a, which was previously considered a diagnostic marker for human hydatid disease and a marker of disease severity, was suppressed by both ESPs and HCF antigens in MSCs. This may be due to species variability or different cell type responses. Other miRNAs, such as miR-124, miR-206-3p, miR-1a-3p, miR-134, miR-23a-3p, miR-199, miR-486-5p, miR-210-3p, and miR-185, showed differential expression after antigenic stimulation and are known to be involved in immune regulation, inflammation, osteogenic differentiation, angiogenesis, and apoptosis (Li et al., 2016; Huang and Zhu, 2018; Zhao et al., 2018, 2023; Chen et al., 2019, 2023; Chen J. X. et al., 2020; Ghafouri-Fard et al., 2021; Yang et al., 2021; Zhang Z. et al., 2021; Wang et al., 2022; Castaño et al., 2023; Dong et al., 2023; Li and Li, 2023; Liu et al., 2023; Ma et al., 2023; Su et al., 2023).

Our analysis of differential miRNAs in different groups revealed interesting findings. The enrichment analysis suggested that ESPs/HCF-intervened MSCs may regulate the RAS–RAF-MAPK pathway, while HCF-intervened MSCs may mediate the mTOR signaling pathway. These pathways play important roles in cell functions such as adhesion, migration, survival, and differentiation (Chen J. et al., 2020; Kodali et al., 2023; Lu et al., 2023; Xiong et al., 2023). Ras proteins, regulated by the MAPK pathway, are critical for bone formation and remodeling (Sharma et al., 2013; Feng et al., 2016; Xue et al., 2023). MSCs EVs have been shown to enhance anti-inflammatory capacity, promote osteoblast function, and regulate macrophage polarization through the MAPK pathway (Xue et al., 2023). Additionally, the ESPs group showed enrichment in the Th17-related pathway, which is involved in inflammatory responses (Kay et al., 2021). MSCs EVs have potential therapeutic value by modulating Th17 cells (Xia et al., 2023). The HCF group target genes were enriched in the HIF-1 pathway, which regulates immunoregulatory functions in MSCs and is associated with chemokine and IL-6 expression (Martinez et al., 2017). The pLL group target genes were enriched in the Wnt signaling pathway, which mediates immunomodulatory effects and improves osteoporosis (Shum et al., 2016; Huang et al., 2021; van Wigcheren et al., 2023; Wang et al., 2023). The pLL group also showed enrichment in focal adhesion, which affects osteogenic differentiation (Zhao et al., 2021).

Our experiments demonstrated that hydatid antigens ESPs and ESPs-EVs diminish the osteogenic capacity of chemokines and MSCs, along with reducing the expression of the osteogenic signature protein ALP. This suggests that certain antigens may have diverse regulatory effects, such as diminishing local innate immunity and osteogenic differentiation capacity, contributing to immune evasion in hydatid disease and indirectly leading to the expansion of hydatid lesions. Previous studies have indicated the physiological significance of chemokine expression in MSCs, as they recruit various immune cells (Fernández-Francos et al., 2021). Neutrophils and macrophages, the first cells recruited around the worm, may exert antiparasitic effects through phagocytosis, degranulation, or the formation of neutrophil extracellular trapping networks (NETs) (Omar and Abdelal, 2023). Alterations in chemokine expression in MSCs may have multiple biological effects and are closely related to lesion development (Yao et al., 2021).

While this study sheds light on the immunomodulatory effects of MSC-derived EVs in response to hydatid antigens, it is imperative to consider certain limitations. The complexity of *in vivo* environments and the potential variability in MSC behavior between humans and the murine model employed in this research may impact the generalizability of our findings. Consequently, additional research incorporating human samples and *in vivo* studies is essential to comprehensively ascertain the implications of our results for the development of treatment strategies for hydatid disease. Although the outcomes observed with ESP-EVs align with some functional enrichment analyses of miRNA target genes in the ESPs group, the intricate composition of EV contents necessitates further investigation to delineate the specific regulatory mechanisms at play. Nonetheless, the identification of differentially expressed miRNAs involved in immune regulation is encouraging, indicating novel potential pathways for interactions between parasites and host cells. By characterizing the altered miRNA expression profiles in EVs of MSCs challenged with hydatid antigens, our study enhances the understanding of the immunomodulatory roles of MSCs in parasitic infections. This advances the groundwork for future research focusing on specific miRNAs in the context of hydatid disease, offering a rationale for targeted studies. In the ELISA experiment, we did not find any changes in the expression of pro-inflammatory or anti-inflammatory cytokines. However, further research may be needed due to differences in cell microenvironment and antigen concentration.

## Conclusion

In conclusion, our study reveals that MSC-derived EVs respond to hydatid antigen stimulation, leading to significant changes in miRNA expression profiles. These alterations impact crucial regulatory pathways involved in immune responses and osteogenic processes. The modulation of miRNA expression by hydatid antigens, particularly ESPs and ESPs-EVs, suggests a potential mechanism for immune evasion by *Echinococcus granulosus*, affecting the progression of hydatid disease. While these antigens reduce osteogenic differentiation, further investigation into the precise molecular mechanisms is needed. The regulation of miRNAs and target genes highlights the therapeutic potential of MSC-derived EVs in hydatid disease treatment. Key regulatory miRNAs and HUB genes, including miR-9-5p, miR-124, miR-23a-3p, Traf1, and Tnf, emerge as potential therapeutic targets for modulating immune responses and osteogenic processes in hydatid disease, offering new avenues for therapeutic interventions against this parasitic infection.

## Data availability statement

The datasets presented in this study can be found in online repositories. The names of the repository/repositories and accession number(s) can be found in the article/Supplementary material.

## Ethics statement

The animal study was approved by laboratory animal ethics committee of first affiliated hospital of Xinjiang Medical University. The study was conducted in accordance with the local legislation and institutional requirements.

## Author contributions

XW: Funding acquisition, Investigation, Methodology, Writing – original draft, Writing – review & editing. WM: Funding acquisition, Project administration, Writing – review & editing. QJ: Data curation, Project administration, Resources, Software, Writing – review & editing. ZY: Formal analysis, Methodology, Writing – review & editing. JM: Methodology, Validation, Writing – review & editing. ZZ: Funding acquisition, Project administration, Writing – review & editing. ZX: Conceptualization, Data curation, Funding acquisition, Investigation, Resources, Supervision, Writing – review & editing.

## Footnotes

^1^ http://www.mirbase.org/

^2^ http://rfam.xfam.org/

^3^ https://www.mdc-berlin.de/content/mirdeep2-documentation

^4^ http://www.microrna.org/microrna/home.do

^5^ https://bibiserv.cebitec.uni-bielefeld.de/rnahybrid

^6^ http://metascape.org

^7^ https://string-db.org/

^8^ https://www.networkanalyst.ca

^9^ https://cytoscape.org/
