# Supplementary figures and images for: Exploration of altered miRNA expression and function in MSC-derived extracellular vesicles in response to hydatid antigen stimulation
Source: Front Microbiol. 2024 Mar 27;15:1381012. doi: 10.3389/fmicb.2024.1381012 (PMC11004373; doi:10.3389/fmicb.2024.1381012)

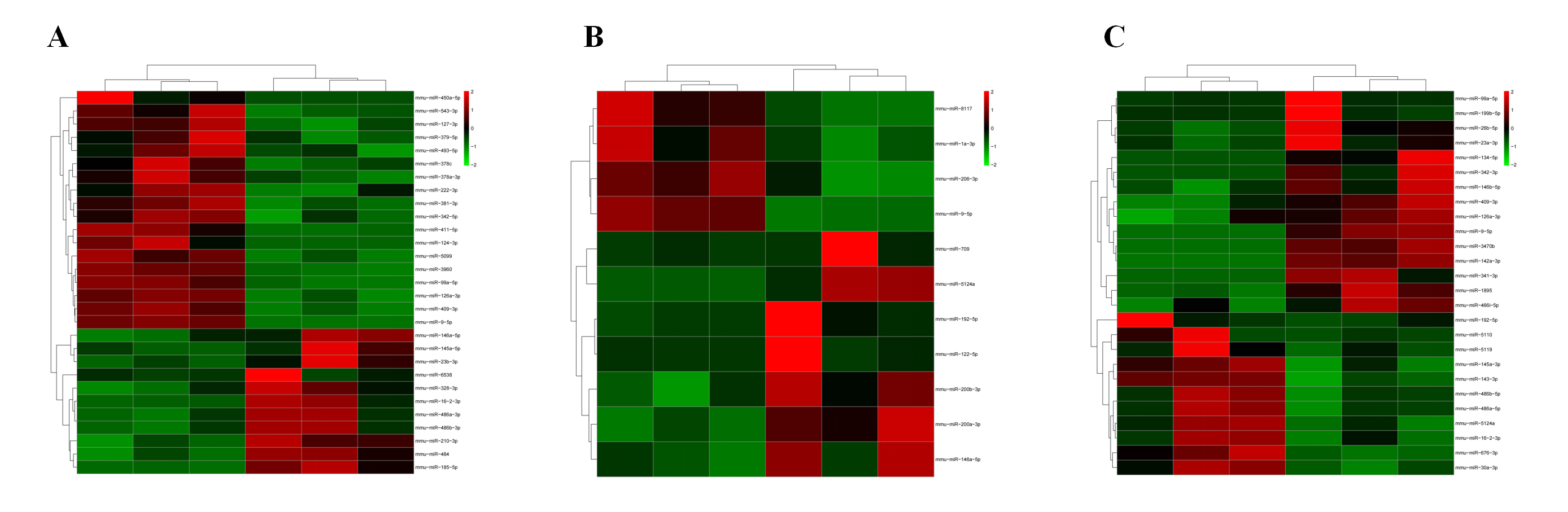

Supplement: Supplementary file 1 [file Image_1.TIF]

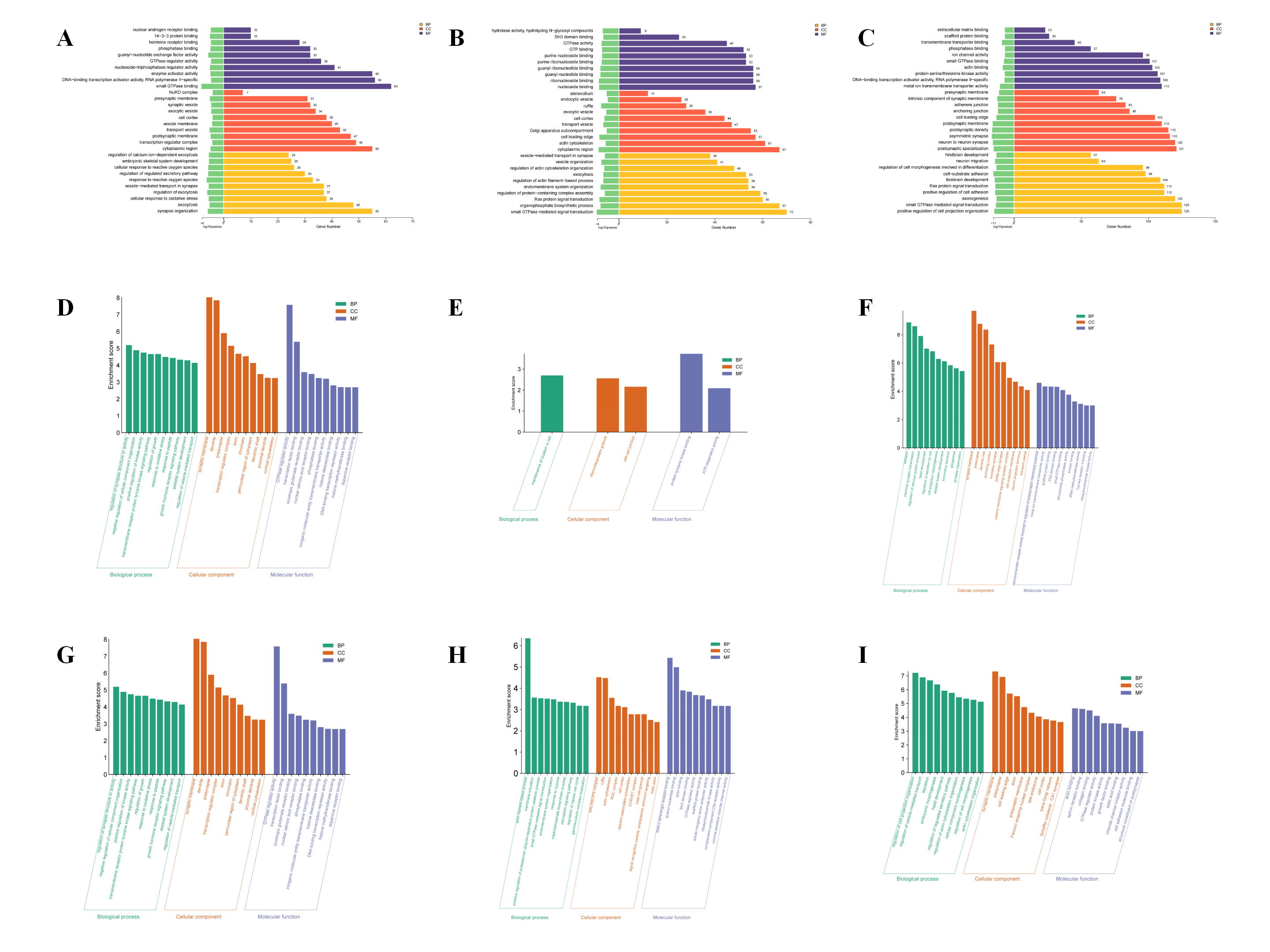

Supplement: Supplementary file 2 [file Image_2.TIF]

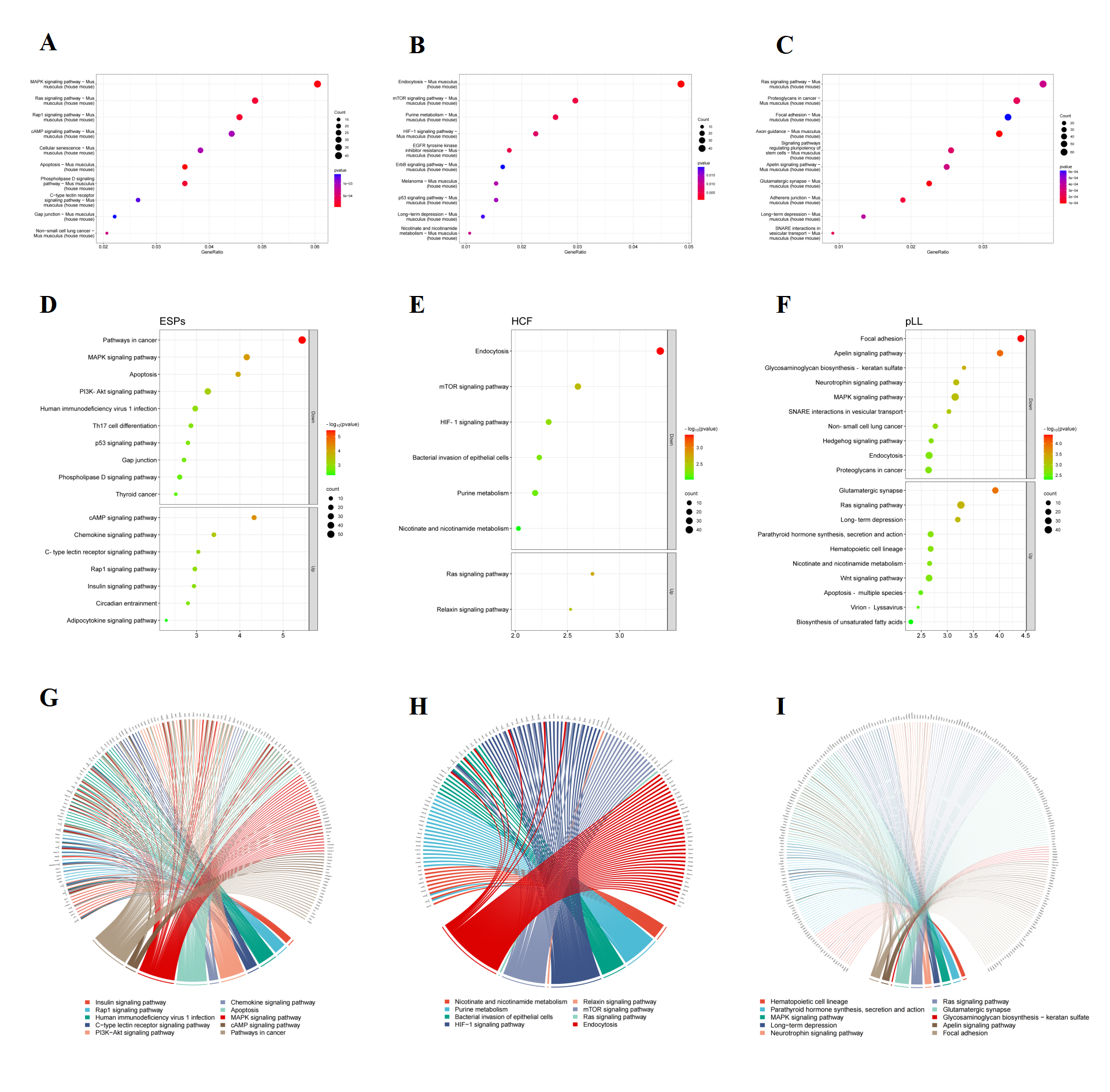

Supplement: Supplementary file 3 [file Image_3.TIF]

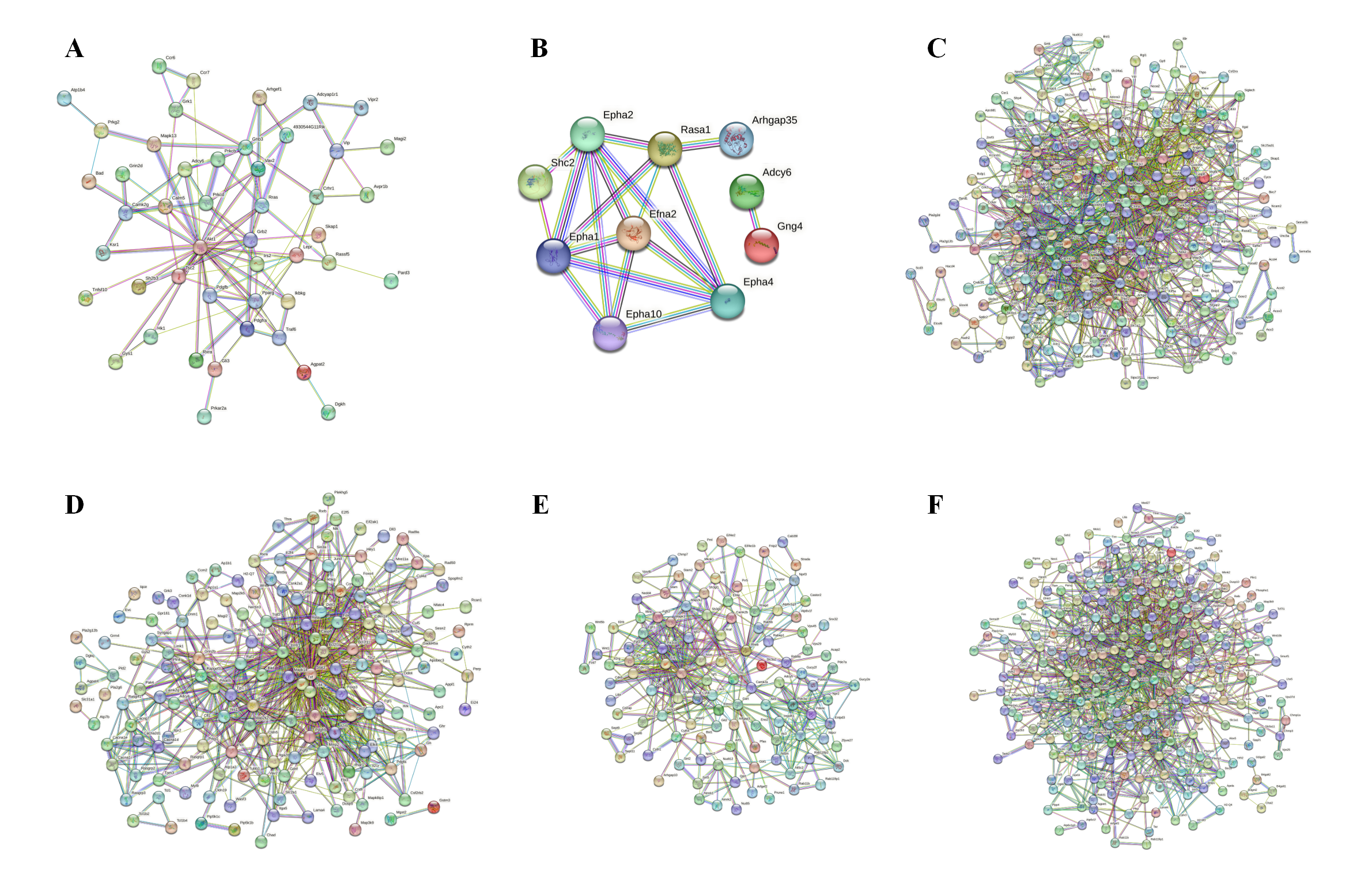

Supplement: Supplementary file 4 [file Image_4.TIF]

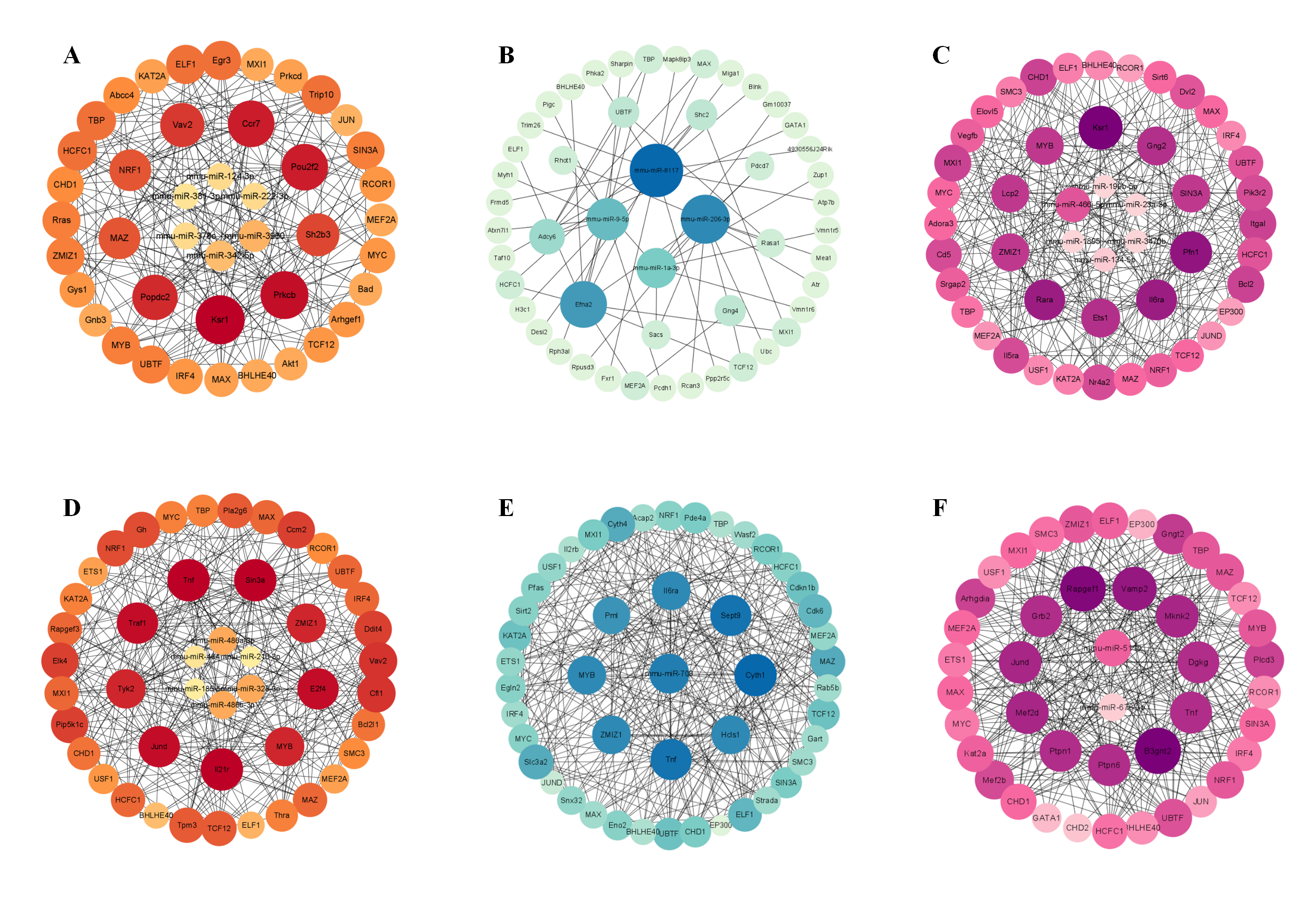

Supplement: Supplementary file 5 [file Image_5.TIF]

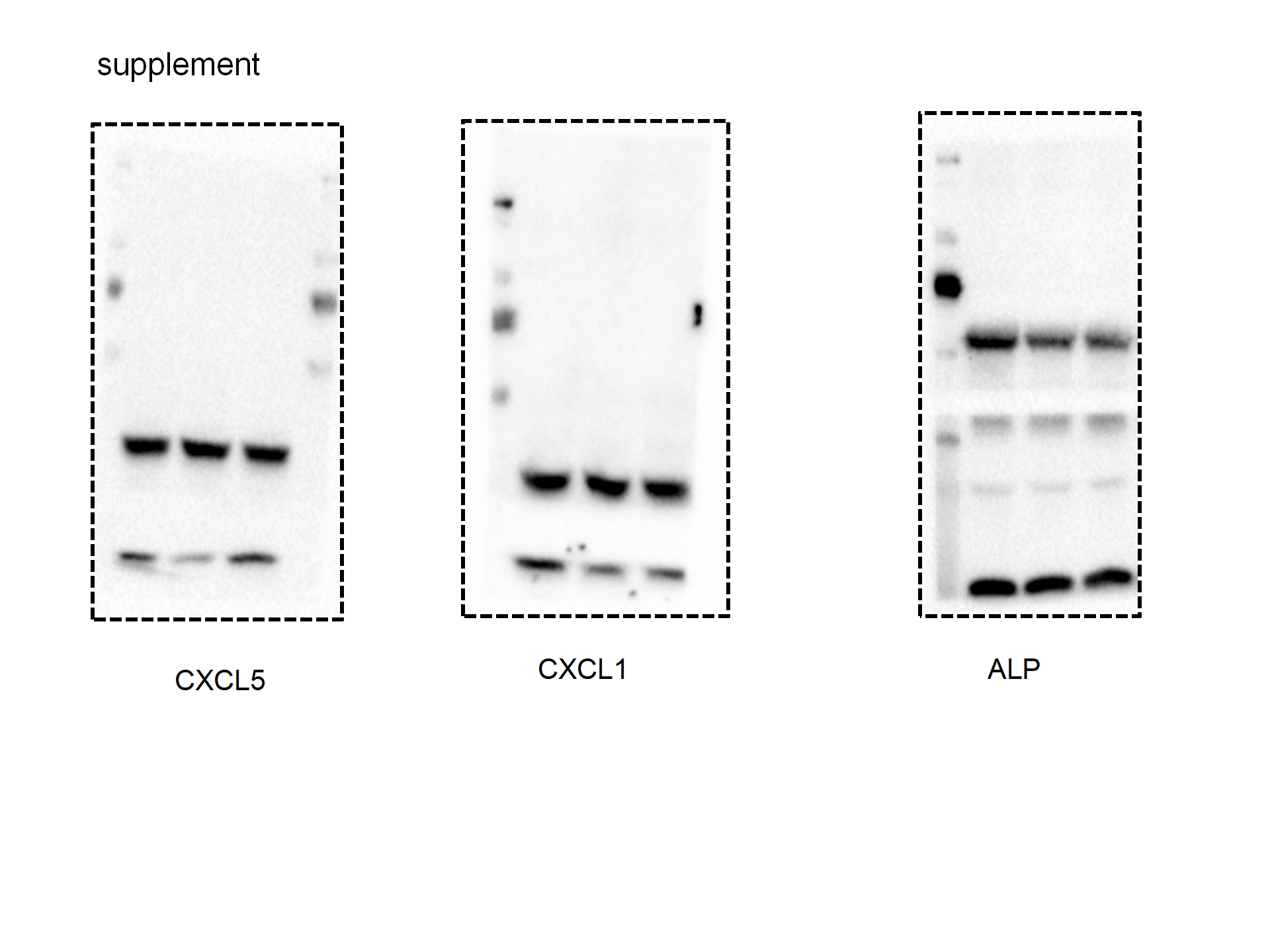

Supplement: Supplementary file 6 [file Image_6.PNG]
